# Supplementary material for: Discovery of van Hove singularities: electronic fingerprints of 3Q magnetic order in a van der Waals quantum magnet
Source: Nat Commun. 2026 Mar 7;17:3610. doi: 10.1038/s41467-026-70063-5 (PMC13096175; doi:10.1038/s41467-026-70063-5)
Supplement: Supplementary file 1 — Supplementary Information [file 41467_2026_70063_MOESM1_ESM.pdf]

Supplementary Materials for

Discovery of Van Hove Singularities: Electronic Fingerprints of 3Q Magnetic Order in a van der Waals Quantum Magnet

Hai-Lan Luo[1,2], Josue Rodriguez[1], Debasis Dutta[3], Maximilian Huber[2], Haoyue Jiang[2,4], Luca Moreschini[1,2], Catherine Xu[1], Alexei Fedorov[5], Chris Jozwiak[5], Aaron Bostwick[5], Guoqing Chang[3], James G. Analystis[1,6,7], Dung-Hai Lee[1,2], and Alessandra Lanzara[1,2,7,*]

[1]*Department of Physics, University of California, Berkeley, Berkeley, CA, USA*

[2]*Materials Sciences Division, Lawrence Berkeley National Laboratory, Berkeley, CA, USA*

[3]*Division of Physics and Applied Physics, School of Physical and Mathematical Sciences, Nanyang Technological University, Singapore, Singapore*

[4]*Graduate Group in Applied Science and Technology, University of California, Berkeley, Berkeley, CA, USA*

[5]*Advanced Light Source, Lawrence Berkeley National Laboratory, Berkeley, CA, USA*

[6]*CIFAR Quantum Materials, CIFAR, Toronto, ON, Canada*

[7]*Kavli Energy NanoScience Institute, University of California, Berkeley, Berkeley, CA, USA*

Supplementary Notes

1. Orbital Symmetries and Polarization Dependence in ARPES

To elucidate the orbital characters of different bands and their visibility in ARPES, we summarize the local crystal-field environment and the corresponding d-orbital splitting. As shown in Fig. S6, the Co^{3+} ions in $Co_{1/3}TaS_2$ reside in a trigonally distorted pseudo-octahedral coordination with D_{3d} crystal-field symmetry, leading to a splitting into $e_2(d_{xy}, d_{x^2-y^2})$, $a_1(d_{z^2})$, and $e_1(d_{xz}, d_{yz})$ levels. In contrast, Ta^{3+} sites possess D_{3h} symmetry, with a splitting into $a_1(d_{z^2})$, $e_2(d_{xy}, d_{x^2-y^2})$, and $e_1(d_{xz}, d_{yz})$ states [1, 2].

In ARPES, the spectral weight is strongly modulated by the dipole matrix element $|M^k_{f,i}|^2 \propto |\langle\phi^k_f|\hat{\varepsilon}\cdot r|\phi^k_i\rangle|^2$, where ϕ^k_i (ϕ^k_f) denotes the initial (final) state wavefunction of the photoelectron, and $\hat{\varepsilon}$ is the unit vector along the polarization direction of the incident light (LH: even parity; LV: odd parity). Approximately, only orbitals sharing the same parity as the incident polarization can be detected in ARPES [3]. When the analyzer slit is aligned along the Γ_0-K_0 direction (parallel to the x-axis as defined in Fig. S6c, the d-orbital symmetries of Co and Ta (a1, e1, and e2 sets) with respect to the scattering plane ($x - z$ plane) are illustrated in Fig. S6(d). The even d_{z^2} orbitals are expected to be visible with LH polarization, whereas the $e1$ (d_{xz}, d_{yz}) and $e2$ ($d_{xy}, d_{x^2-y^2}$) sets are not collectively symmetric with respect to the scattering plane and may appear under both LH and LV polarizations. Accordingly, the observed bands can be classified into three categories: (1) the γ_K and γ_M bands, visible only under LH polarization and thus mainly derived from d_{z^2} orbitals; (2) the α and β bands, visible under both LH and LV polarizations but significantly stronger in LH, which can arise from all orbitals but are dominated by d_{z^2} character; and (3) the ϵ band, which appears predominantly under LV polarization, is mainly contributed by d_{xz}/d_{yz} and $d_{xy}/d_{x^2-y^2}$ sets.

Together with the DFT-calculated band structures projected onto individual Co and Ta orbitals (see Fig. S7), the above polarization-dependent analysis can be further extended. The DFT results enable a clear separation of Co- and Ta-derived contributions, indicating that the near-E_F γ_K and γ_M bands mainly arise from Co $3d_{z^2}$ orbital; whereas the highly-dispersive α and β bands are dominated by Ta $5d_{z^2}$ and Ta $5d_{xy}/d_{x^2-y^2}$ states.

2. Temperature-Dependent Measurements

We performed temperature-dependent ARPES measurements on $Co_{0.31}TaS_2$ along the K-M-K' direction (Fig. S14). As shown in Fig. S14a, at 6 K within the 3**Q** ordered state, the near-E_F band exhibits an inverse Mexican-hat-like dispersion. The energy distribution curves (EDCs) obtained at M (k1) and k2 (Fig. S14d) show that the peak position of EDC(k1) is located at a slightly yet clearly higher binding energy than that of EDC(k2). This behavior is consistent with a 3**Q**-order-induced reconstruction that produces the inverse Mexican-hat-like dispersion described in the main text.

By contrast, at 31 K (1**Q** order) and 82 K (paramagnetic state), the dispersions in Fig. S14b and S14c no longer exhibit the inverse Mexican-hat-like structure. Instead, the EDC peak positions at k1 and k2 (Fig. S14e and S14f) are nearly identical, indicating a hole-like band dispersion along K-M-K'. Taken together, these results demonstrate that the inverse Mexican-hat-like dispersion emerges exclusively in the 3**Q** phase, while the 1**Q** and paramagnetic phases show a hole-like band. This provides direct spectroscopic evidence that the 3**Q** magnetic order strongly modifies the low-energy electronic structure.

3. Temperature-Dependent Simulated EDCs

To clarify whether the Mexican-hat-like dispersion can be washed out by thermal broadening at 82 K, we performed quantitative simulations that incorporate two sources of thermal broadening: the Fermi-Dirac cutoff and the temperature evolution of the intrinsic scattering rate. To model the ARPES spectra, we start from an intrinsic spectral peak represented by a Lorentzian $L(E)$ centered at $E - E_F = E_0$ with a full width at half maximum (FWHM) Γ. The temperature-dependent occupation is introduced by multiplying this spectrum by the Fermi-Dirac distribution $f(E,T)$. The resulting curve is then convolved with the experimental energy resolution, which is modeled as a Gaussian $G(E;\Delta E_{res})$ with FWHM ΔE_{res}. The simulated EDC is therefore

$$I_{\mathrm{sim}}(E,T) = [L(E)f(E,T)] \otimes G(E), \tag{1}$$

where

$$L(E) = \frac{1}{\pi}\frac{\Gamma/2}{(E-E_0)^2 + (\Gamma/2)^2}, \tag{2}$$

$$f(E,T) = \frac{1}{\exp\left(\frac{E-E_F}{k_B T}\right) + 1}, \tag{3}$$

and

$$G(E;\Delta E_{\text{res}}) = \frac{1}{\sigma\sqrt{2\pi}}\exp\left(-\frac{E^2}{2\sigma^2}\right), \qquad \sigma = \frac{\Delta E_{\text{res}}}{2\sqrt{2\ln 2}}, \tag{4}$$

Here, k_B is the Boltzmann constant, and $\otimes$ denotes convolution [3].

To simulate the 6 K spectra, as shown in Fig. S14g, we set $T = 6\,\text{K}$, $\Delta E_{\text{res}} \approx \sqrt{\Delta E^2_{\text{analyzer}} + \Delta E^2_{h\nu}} = 10.6$ meV , and $\Gamma = 24\,\text{meV}$, and choose $E_0 = -6\,\text{meV}$ and $-2\,\text{meV}$ to best reproduce the experimental EDC(k1) and EDC(k2) from Fig. S14d, respectively. The inverse Mexican-hat-like dispersion is reflected in the higher binding energy of the EDC($-6\,$meV) peak relative to the EDC($-2\,$meV) peak. To examine whether increasing temperature could smear out the Mexican-hat feature, we then simulate the 31 K and 82 K spectra (Fig. S14h and S14i). In these simulations, the peak center energies E_0 are kept fixed at $-6\,$meV and $-2\,$meV for the orange and blue curves, respectively, and only two physically motivated quantities are varied: (i) the temperature in the Fermi-Dirac distribution, and (ii) the Lorentzian FWHM Γ, which is adjusted to best reproduce the experimental data. The latter is allowed to increase modestly with temperature because the intrinsic linewidth reflects the imaginary part of the self-energy (i.e., the intrinsic scattering rate), which generally grows with temperature due to electron-phonon and electron-electron interactions.

Importantly, these simulations show that over the temperature range studied (6 - 82 K), even after including both sources of thermal broadening, an inverse Mexican-hat-like dispersion that persists to high temperature would still produce a clearly deeper EDC peak for $E_0 = -6\,$meV than for $E_0 = -2\,$meV. Thermal broadening alone therefore cannot hide such an inverse Mexican hat-like dispersion within our experimental temperature window. In contrast, the 82 K data (Fig. S14f) exhibit the opposite behavior: the EDC(k1) peak becomes slightly shallower than the EDC(k2) peak. Thus, the comparison between panels (d-f) and (g-i) shows that the hole-like dispersion observed at 31 K and 82 K cannot be generated by thermal broadening of an inverse Mexican-hat-like band and is instead intrinsic. Notably, stronger thermal broadening could, in principle, smear out this characteristic inverse Mexican-hat-like band. As illustrated by the example in the inset of Fig. S14i, when the temperature is raised to 200 K and the FWHM is increased to Γ=100 meV, the two EDCs nearly coincide, such that the underlying inverse Mexican-hat-like band becomes effectively indiscernible.

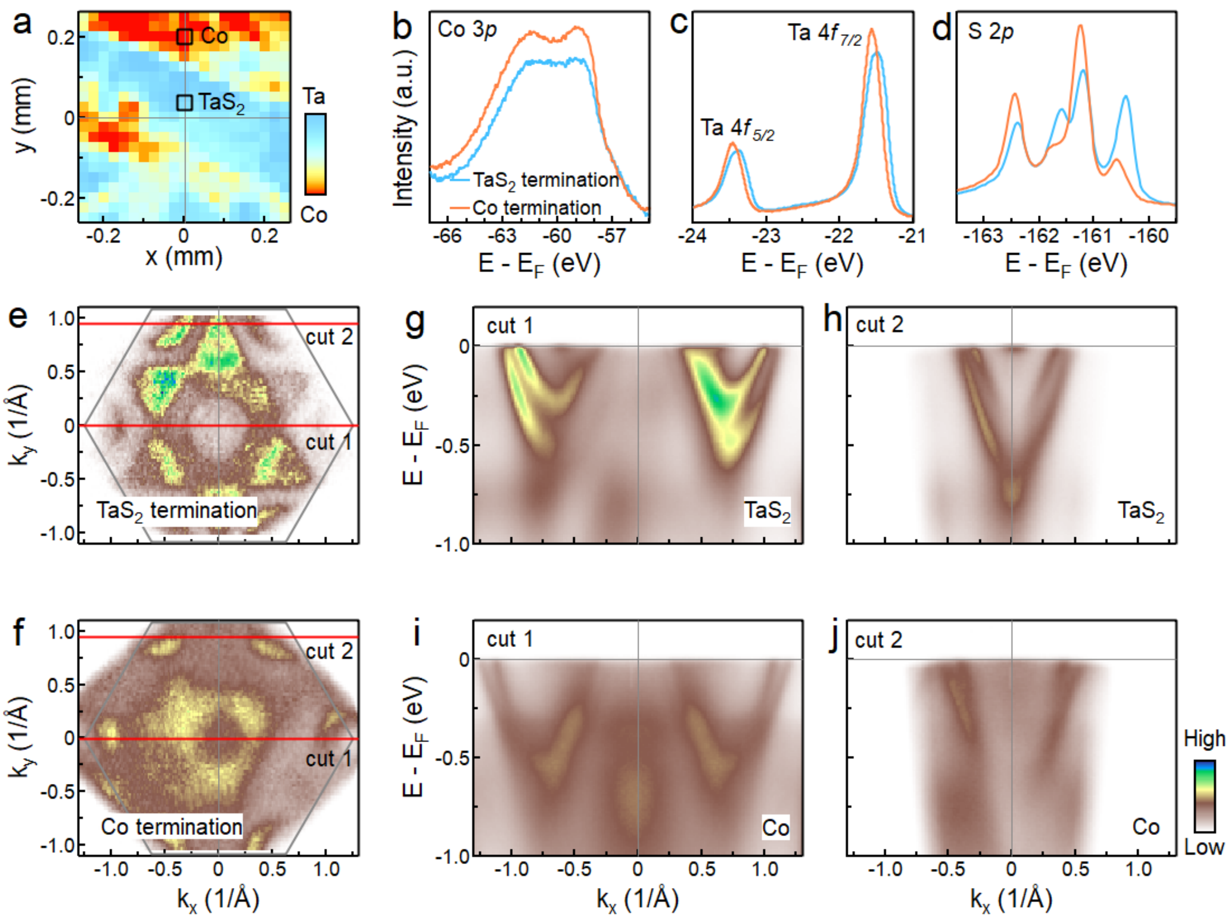

Fig. S 1: **Distinct electronic structures on two surface terminations of $Co_{0.32}TaS_2$. a** Spatially resolved X-ray photoelectron spectroscopy (XPS) map showing the integrated spectral weight of the S 2p core level in the energy range $E - E_F = -160 \sim -160.8$ eV. **b-d** Core-level spectra corresponding to the TaS_2 (blue) and Co (orange) surface terminations, measured at $T = 7$ K with photon energy $h\nu = 200$ eV. The spectra were extracted from the respective regions marked in (**a**). **e-f** Fermi-surface maps acquired from the TaS_2 (**e**) and Co (**f**) surface terminations, corresponding to the areas highlighted in (**a**). **g-h** Band structures of the TaS_2-terminated surface measured along the directions labeled "cut1" (**g**) and "cut2" (**h**), as marked by red lines in (**e**). **i-j** Band structures of the Co-terminated surface measured along the directions labeled "cut1" (**i**) and "cut2" (**j**), as indicated by red lines in (**f**).

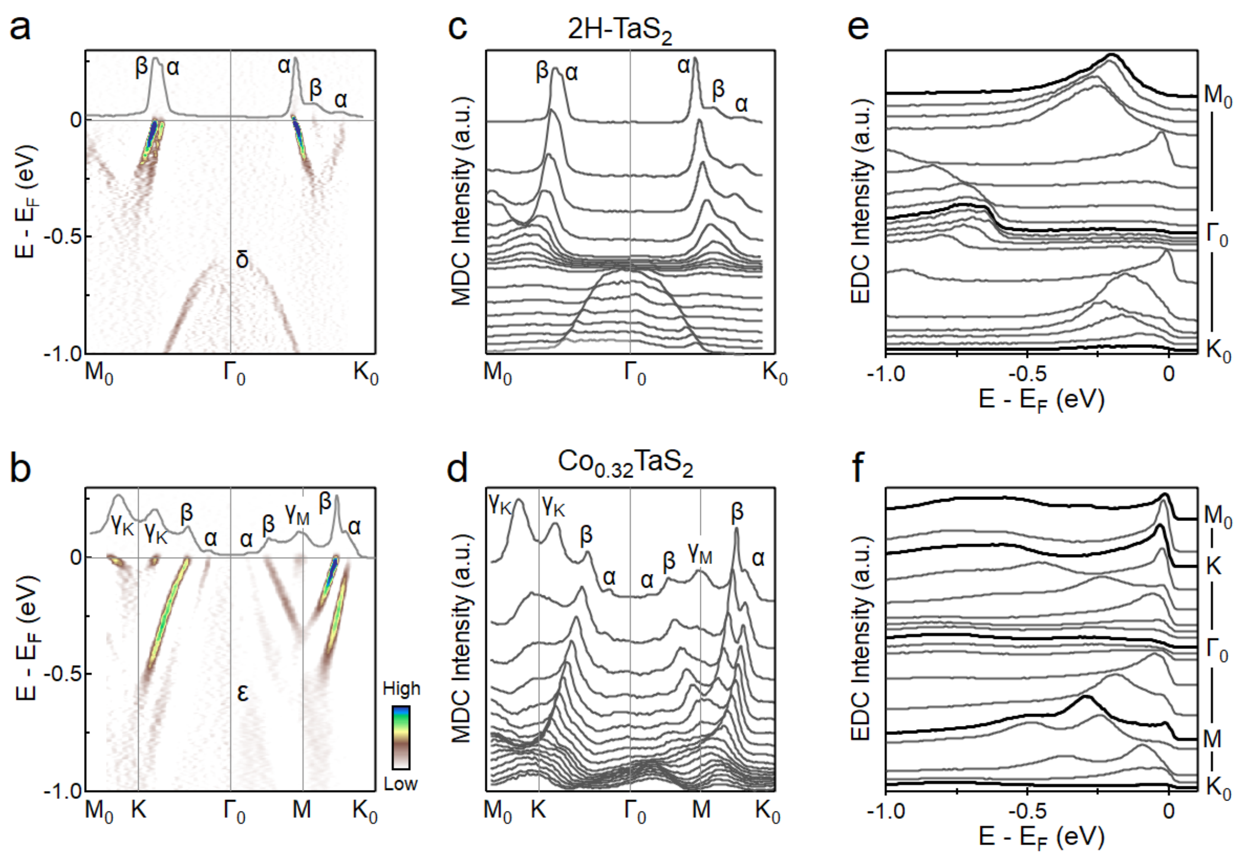

Fig. S 2: **Momentum distribution curve (MDC) and energy distribution curve (EDC) stacks obtained along** M_0-Γ_0-K_0 **in 2**H**-TaS**$_2$ **and Co**$_{0.32}$**TaS**$_2$**. a-b** Second-derivative images with respect to momentum along the M_0-Γ_0-K_0 direction for $2H$-TaS_2 (**a**) and $Co_{0.32}TaS_2$ (**b**). The MDCs obtained at the Fermi level from the raw data in Figure 1g and 1h are overlaid on the respective images. **c-d** MDC stacks obtained along the M_0-Γ_0-K_0 direction from the raw data shown in Figure 1g for $2H$-TaS_2 (**c**) and Figure 1h for $Co_{0.32}TaS_2$ (**d**). **e-f** EDC stacks obtained along the M_0-Γ_0-K_0 direction from the raw data shown in Figure 1g for $2H$-TaS_2 (**e**) and Figure 1h for $Co_{0.32}TaS_2$ (**f**). EDCs at high-symmetry momenta (M_0, K, Γ_0, M, and K_0) are highlighted with black lines.

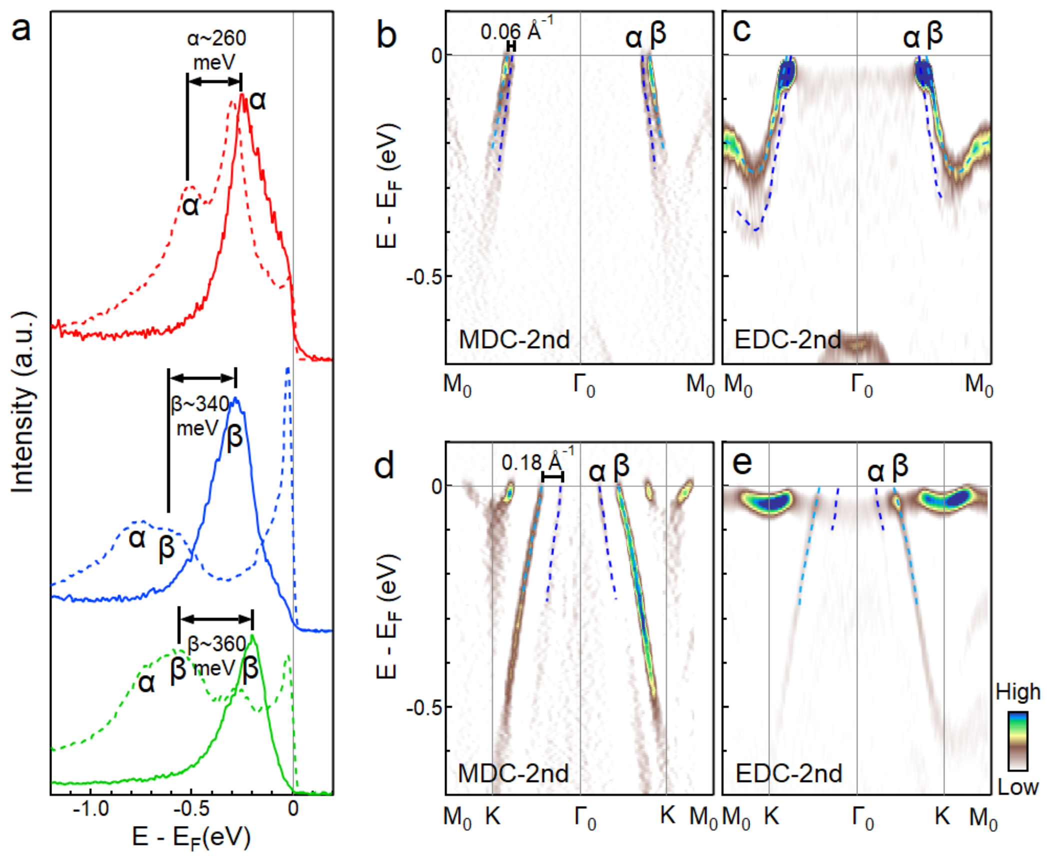

Fig. S 3: **Band-selective doping in $Co_{0.32}TaS_2$ due to Co intercalation and α-β band splitting in 2*H*-TaS_2 and $Co_{0.32}TaS_2$.** **a** Comparison of EDCs obtained through the band bottoms and at high-symmetry momenta between 2*H*-TaS_2 and $Co_{0.32}TaS_2$. The red solid and dashed curves represent EDCs through the band bottom along the Γ_0-K_0 direction for 2*H*-TaS_2 (Figure 1g) and $Co_{0.32}TaS_2$ (Figure 1h), respectively. The blue solid and dashed curves are obtained through the band bottom along Γ_0-M_0 for 2*H*-TaS_2 (Figure 1g) and $Co_{0.32}TaS_2$ (Figure 1h), respectively. The green solid and dashed curved are obtained at the M_0 point for 2*H*-TaS_2 (Figure 1g) and $Co_{0.32}TaS_2$ (Figure 1h), respectively. Relative to 2*H*-TaS2, the band bottom in $Co_{0.32}TaS_2$ shifts downward by approximately 260 meV along Γ_0-K_0 and 340 meV along Γ_0-M_0, and the band structure at M_0 shifts downward by ∼360 meV. **b-c** Band structures of 2*H*-TaS_2 measured along the M_0-Γ_0-M_0 high-symmetry direction. These are the second-derivative images with respect to momentum (**b**) and energy (**c**), obtained from the data in Figure 1g. The dark blue and light blue dashed curves are visual guides for the α and β bands. **d-e** Same as (**b**) and (**c**), but for $Co_{0.32}TaS_2$, based on the data in Figure 1h. The momentum separation between the α and β bands at the Fermi level is approximately 0.06 $Å^{-1}$ in 2*H*-TaS_2 and 0.18 $Å^{-1}$ in $Co_{0.32}TaS_2$.

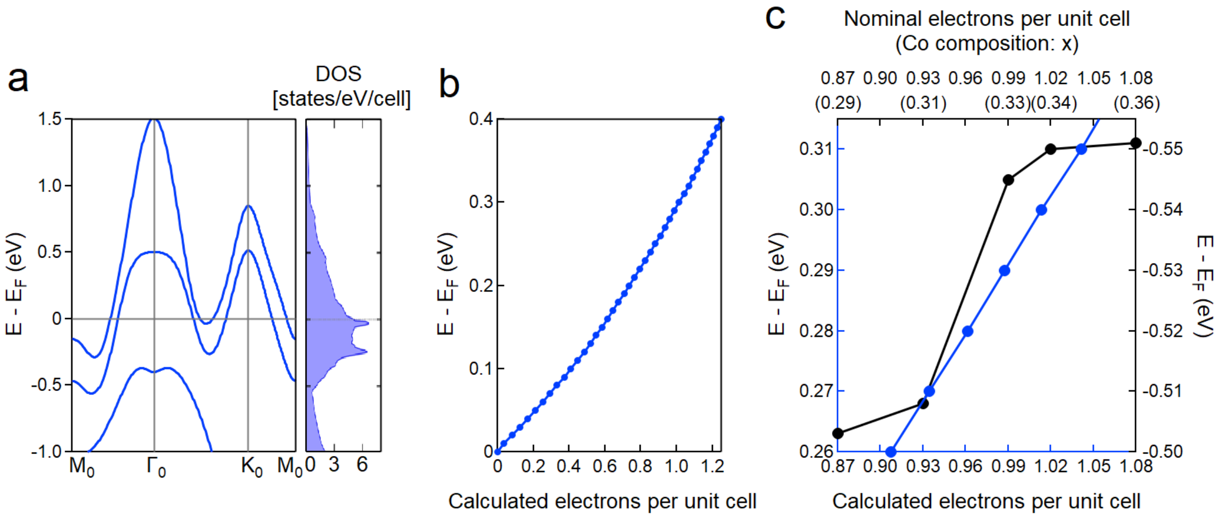

Fig. S 4: **Calculated and experimental electron-doping dependence of TaS_2 bands. a** Calculated band structure of 2H-TaS_2 along the M_0 - Γ_0 - K_0 - M_0 direction. The right panel displays the calculated density of states (DOS) integrated over the entire Brillouin zone as a function of energy. **b** Calculated relation between the number of additional electrons per TaS_2 unit cell and the upward shift of the Fermi level. **c** Comparison between experiment and calculation: the black curve represents the evolution of the α-band minimum along the Γ_0 - K_0 direction as a function of the nominal electron count per TaS_2 unit cell ($3x$, taken from Figure 2e), while the blue curve shows the calculated relation between electron doping and Fermi-level shift.

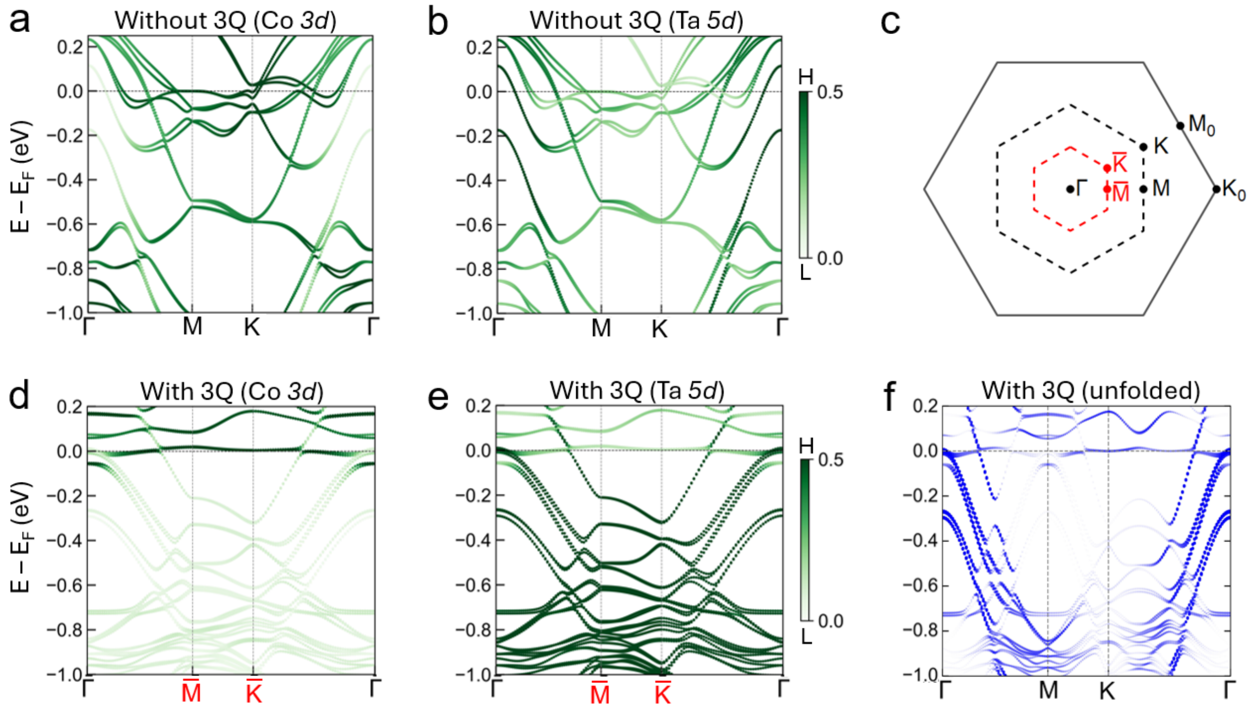

Fig. S 5: **DFT band structures of $Co_{1/3}TaS_2$ without and with 3Q magnetic order. a-b** Calculated bulk band structures of $Co_{1/3}TaS_2$ without 3**Q** magnetic order, projected onto the Co 3*d* (**a**) and Ta 5*d* (**b**) orbitals. **c** Brillouin zones of 2H-TaS_2 (solid hexagon), $Co_{1/3}TaS_2$ without 3**Q** order (black dashed hex-agon), and $Co_{1/3}TaS_2$ with 3**Q** order (red dashed hexagon). **d-e** Calculated bulk band structures of $Co_{1/3}TaS_2$ with 3**Q** magnetic order, projected onto the Co 3*d* (**d**) and Ta 5*d* (**e**) orbitals. **f** DFT band structure of $Co_{1/3}TaS_2$ with 3**Q** magnetic order, unfolded into the Brillouin zone of the non-magnetic $Co_{1/3}TaS_2$.

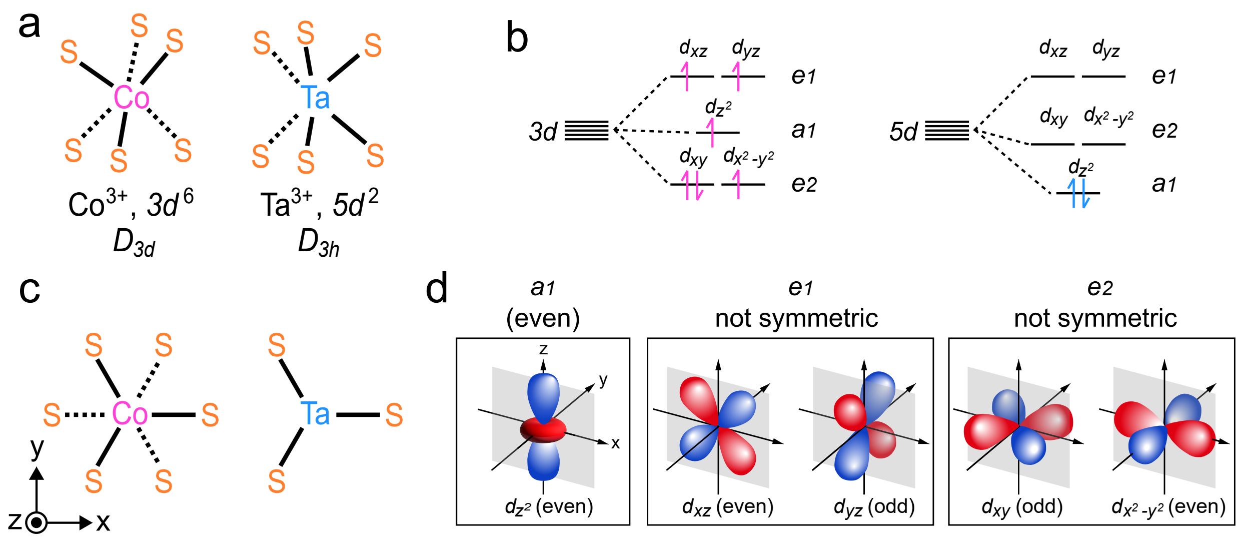

Fig. S 6: **Orbital symmetries and polarization dependence in ARPES.** **a** Local coordination environments of Co and Ta centers and their surrounding S atoms in $Co_{1/3}TaS_2$. **b** Qualitative diagrams of d-orbital splitting for isolated Co and Ta centers under their respective local ligand fields. **c** Real-space projections on the x-y plane showing the local coordination geometries for Co and Ta. **d** Symmetries of the d orbitals for Co and Ta with respect to the scattering plane, defined as the x-z plane aligned along the Γ_0 - K_0 direction [2].

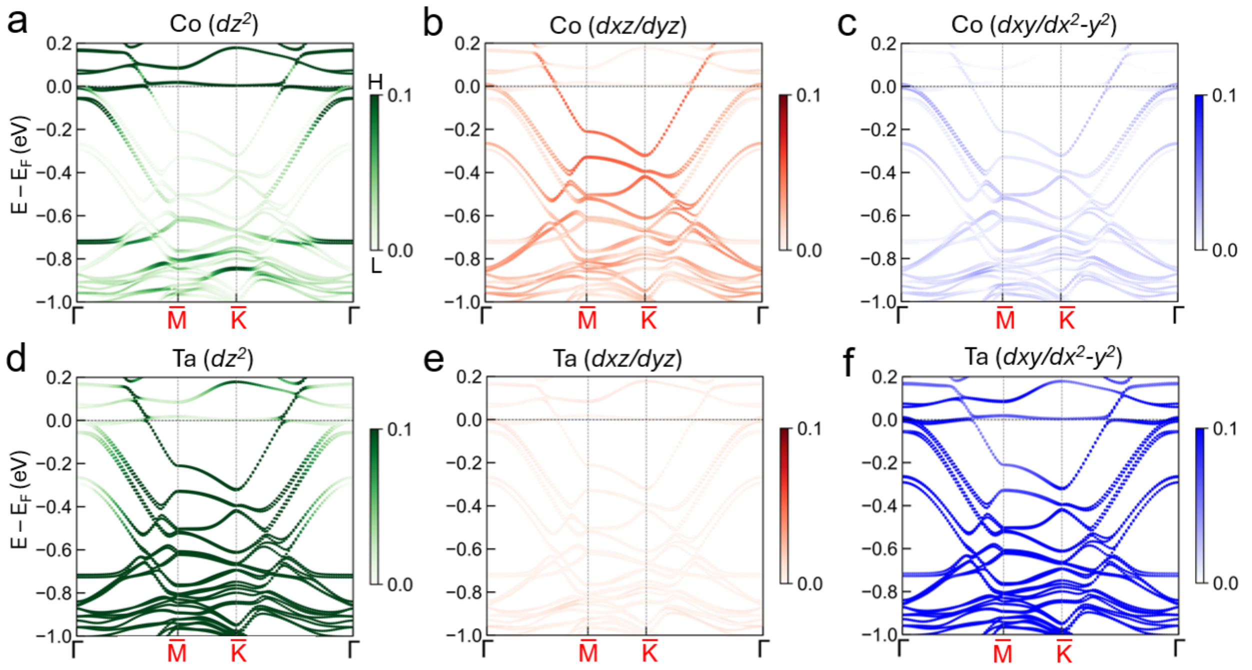

Fig. S 7: **DFT-calculated orbital-projected band structures of bulk $Co_{1/3}TaS_2$ with 3Q magnetic order. a-c** Calculated band structures of $Co_{1/3}TaS_2$ with 3**Q** order, projected onto Co d_{z^2} (**a**), Co d_{xz}/d_{yz} (**b**), Co $d_{xy}/d_{x^2-y^2}$ (**c**) orbitals. **d-f** Corresponding orbital projections onto Ta d_{z^2} (**d**), Ta d_{xz}/d_{yz} (**e**), Ta $d_{xy}/d_{x^2-y^2}$ (**f**) orbitals. The color scale represents the orbital-weight intensity.

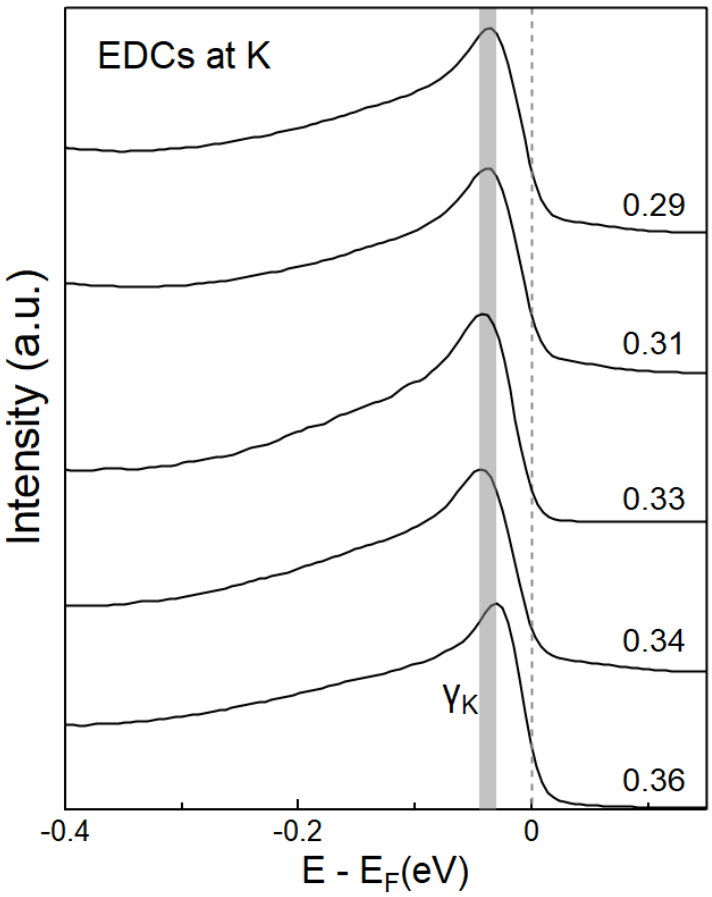

Fig. S 8: **Composition-dependent EDCs of Co_xTaS_2 ($x = $ 0.29, 0.31, 0.33, 0.34, and 0.36) measured at K.** The shaded region indicates the peak position at the lowest doping level (x=0.29).

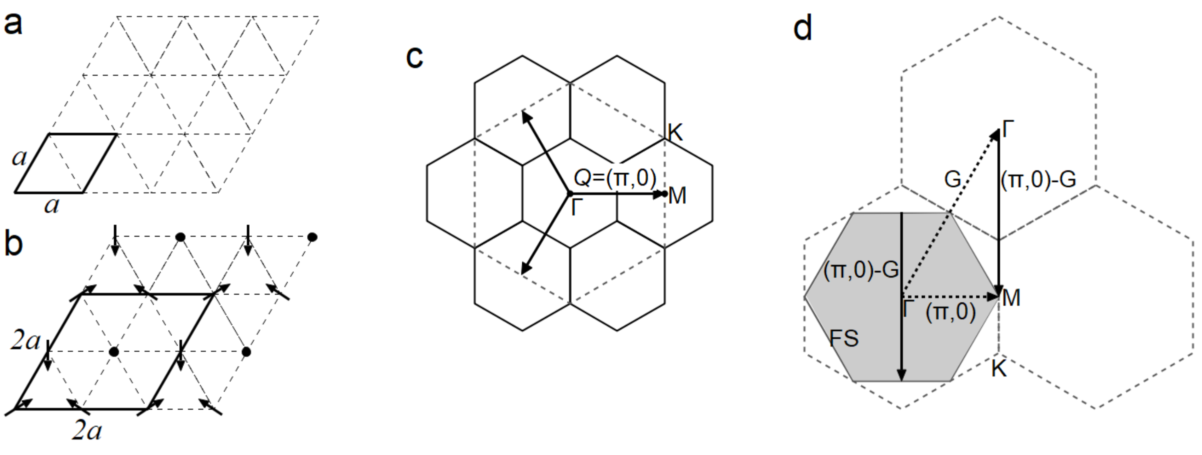

Fig. S 9: **The 3Q magnetic order in real space and the reconstructed Brillouin zone. a** The triangular lattice of intercalated Co atoms (indicated by gray dashed lines) and its unit cell (outlined by black lines). **b** Configuration of the **3Q** magnetic order with four sublattices and the resultant 2×2 magnetic unit cell (black lines). **c** The Brillouin zone of the Co triangular lattice, shown both without (gray dashed lines) and with (black solid lines) the **3Q** magnetic order. The arrow indicates the folding wave vector (π,0). **d** Nesting wave vector ((π,0)-G) that connects the edge centers of the hexagonal Fermi surface at 3/4-filling. The nesting vector ((π,0)-G) is equivalent to (π, 0) up to a reciprocal lattice vector **G**.

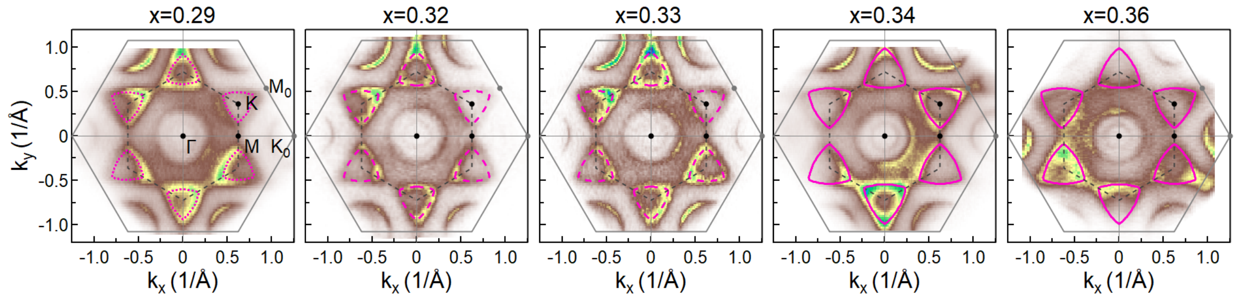

Fig. S 10: **Experimentally extracted Fermi pocket γ as a function of Co composition x.** The dotted ($x = 0.29$), dashed ($x = 0.32, 0.33$), and solid ($x = 0.34, 0.36$) curves represent the experimentally extracted γ Fermi pockets. These curves, which serve as guides to the eye, are derived by quantifying the peak positions of the MDCs obtained from cuts taken parallel to both the Γ–M and Γ–K directions.

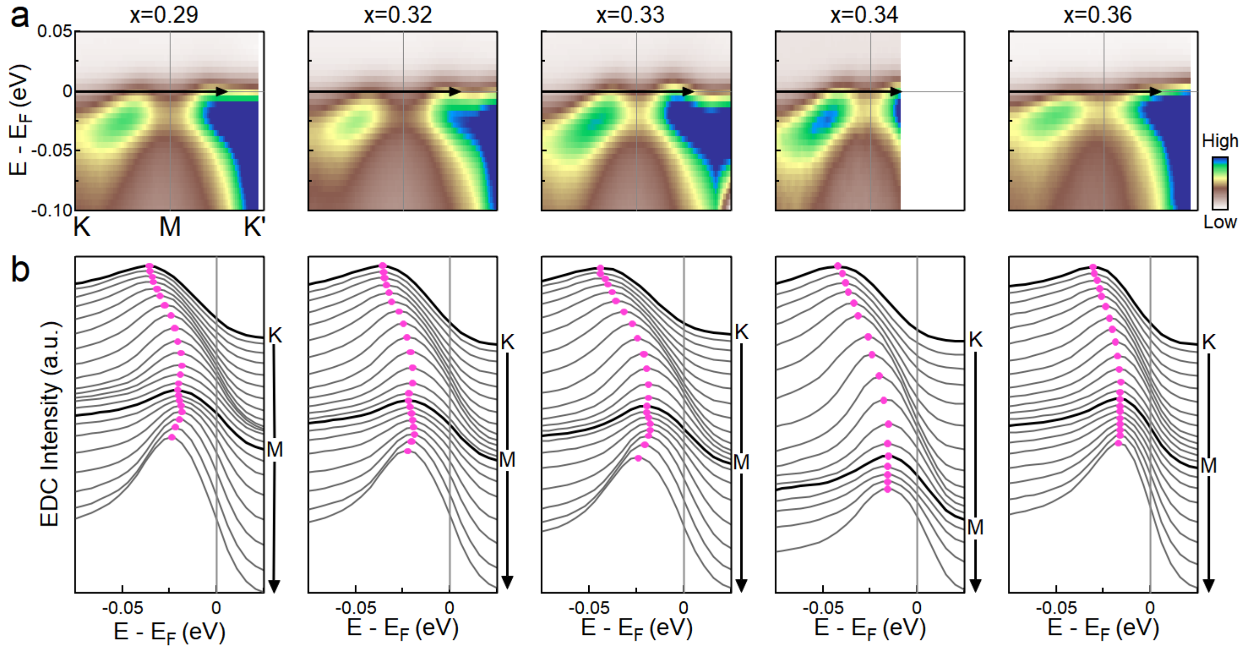

Fig. S 11: **Inverse Mexican-hat-like band dispersions.** **a** Band structure of Co_xTaS_2 along the K-M-K′ direction for varying x. **b** EDC stacks extracted along the K-M direction. The peak positions of each EDC are marked by dots. For the $x = 0.29$, 0.32, and 0.33 samples, band dips appear at the M point, located approximately 4 meV below the band tops. These dips disappear in the $x = 0.34$ and 0.36 samples. For $x = 0.33$, although transport measurements no longer show an anomalous Hall effect, a remnant dip at M remains visible in ARPES, which may originate from short-range magnetic order or spatial inhomogeneity near the phase boundary.

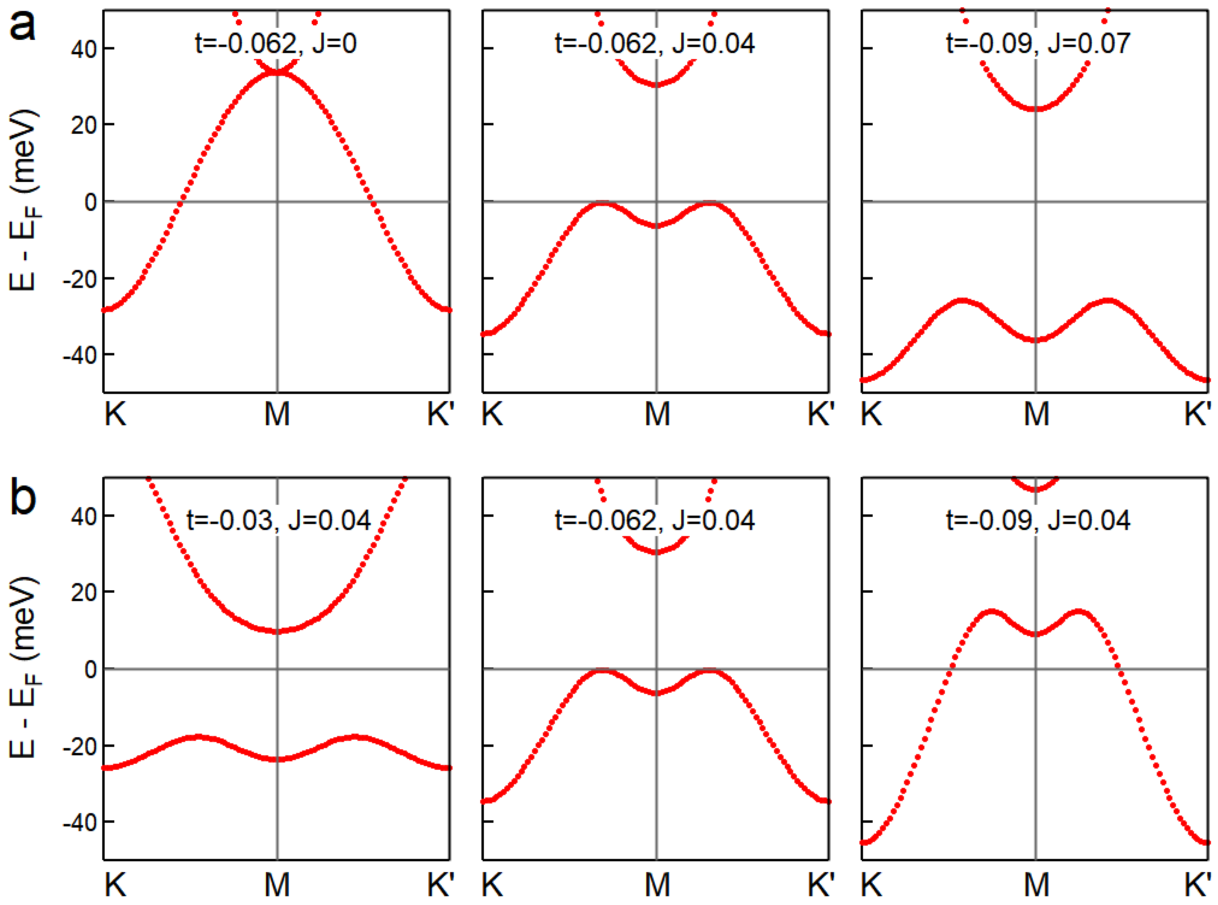

Fig. S 12: **Sensitivity of the band structures near the M point to variations of the hopping amplitude t and exchange coupling J. a** Band structures calculated for different values of t at a fixed $J = 0.04$. The results show that a finite exchange coupling J induces the emergence of an inverse Mexican-hat-like dispersion around the M point. **b** Band structures calculated for different values of J at a fixed $t =$ -0.062. Varying t and J mainly affects the bandwidth and the energy separation between the M-point minimum and the neighboring maxima. All calculations were performed with the chemical potential $\mu = 2.545\,t$.

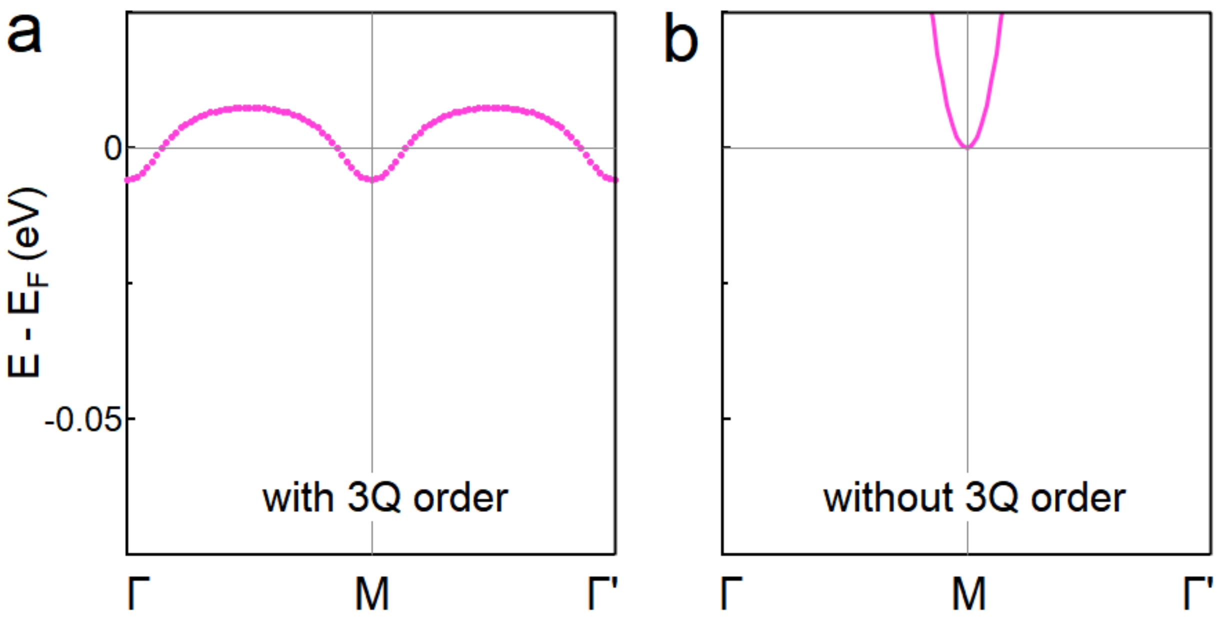

Fig. S 13: **Calculated band dispersions along Γ-M-Γ′.** **a** Calculated band dispersion along Γ-M-Γ′ for the triangular lattice with the 3**Q** magnetic order at a band filling slightly deviating from 3/4. **b** Calculated band dispersion along Γ-M-Γ′ for the triangular lattice without the 3**Q** magnetic order at 3/4-filling. The computational parameters used in these calculations are provided in the Methods section.

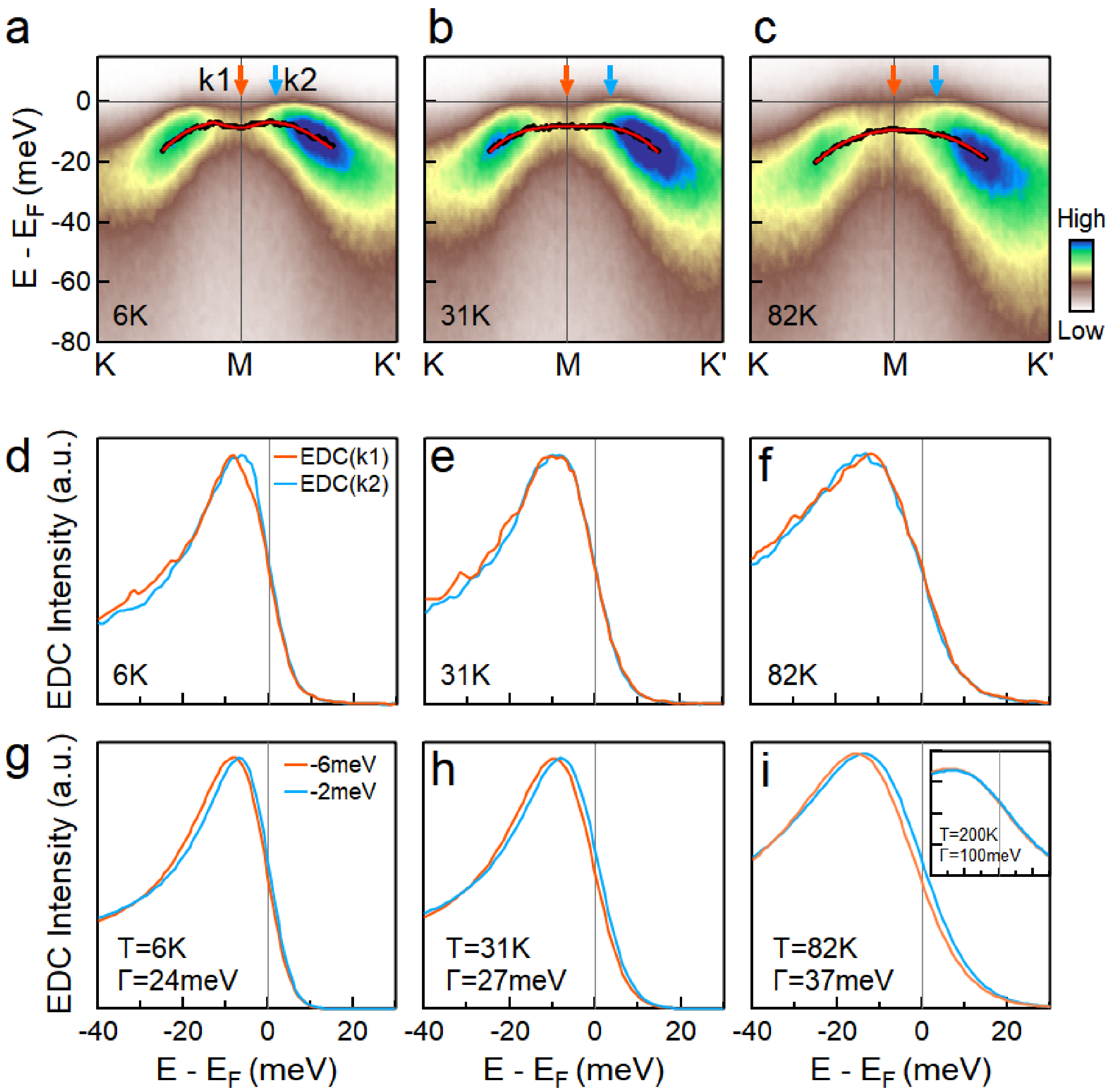

Fig. S 14: **Temperature-dependent ARPES spectra of $Co_{0.31}TaS_2$ along the K-M-K$'$ direction.** **a-c** Band structures measured at 6 K, 31 K and 82 K, respectively. Arrows mark the momenta k1 (M point) and k2. **d-f** EDCs extracted at k1 and k2 from (**a-c**). **g-i** Simulated EDCs at 6 K, 31 K, 82 K, and 200 K (inset of (**i**)), assuming that the underlying band dispersion remains the same inverse Mexican-hat-like dispersion as at 6 K; i.e., all orange and blue curves generated from a Lorentzian lineshape $L(E)$ centered at $E_0 = -6$ meV and -2 meV, respectively. The FWHM value Γ used for simulations at 6 K, 31 K, and 82 K is chosen to best reproduce the experimental EDCs in (**d-f**).

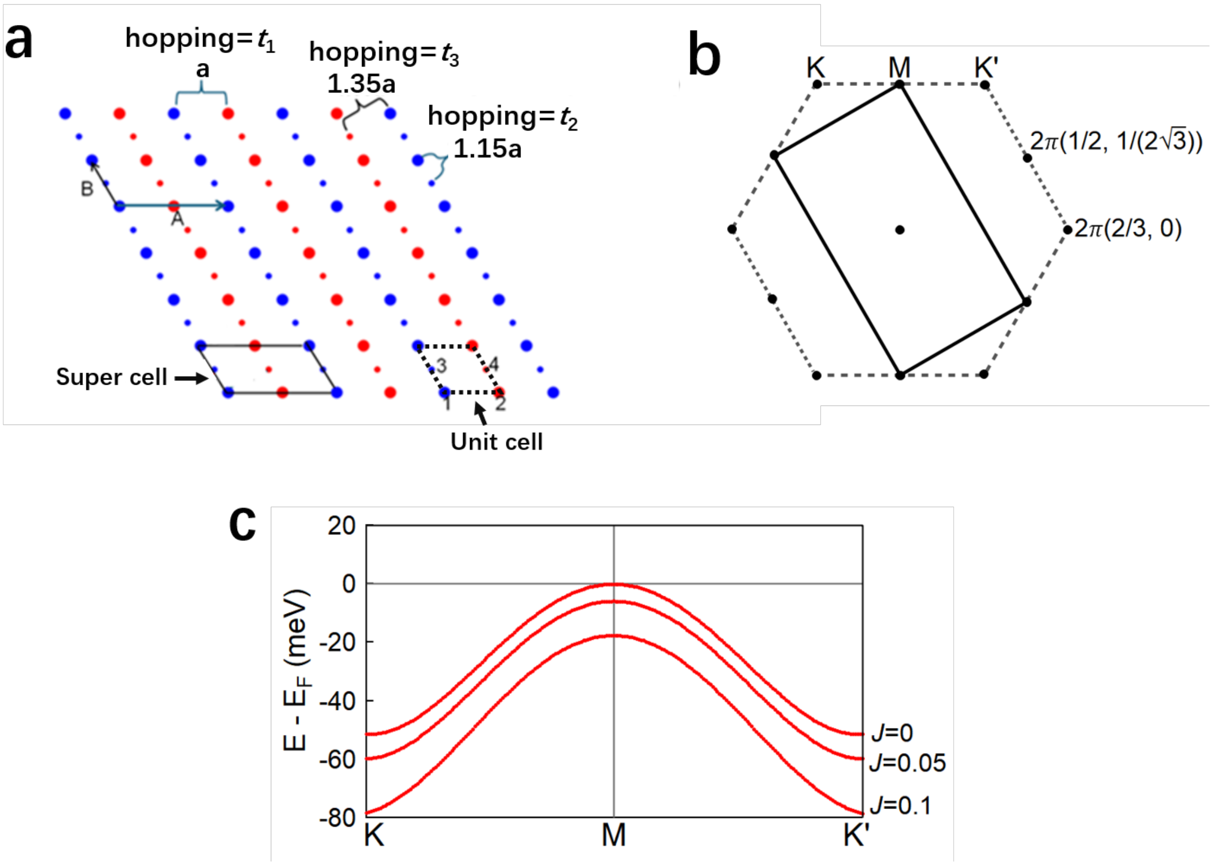

Fig. S 15: **Band structure of the 1Q ordered state.** **a** Schematic illustration of the 1**Q** ordered structure with two adjacent layers. The hopping parameters t_1, t_2, and t_3 are assigned according to the bond lengths, and the supercell (2a×a) is shown in comparison with the unit cell (a×a). **b** Brillouin zones of the unit cell (a×a) without 1**Q** order and of the supercell (2a×a) with 1**Q** order, showing the high-symmetry K-M-K′ path. **c** Band dispersions calculated from the tight-binding Hamiltonian on the 2a×a lattice for different values of J with fixed $t_1 = 0.08$, $t_2 = $-0.04, $t_3 = $-0.005, and μ=-2.85 t_1.

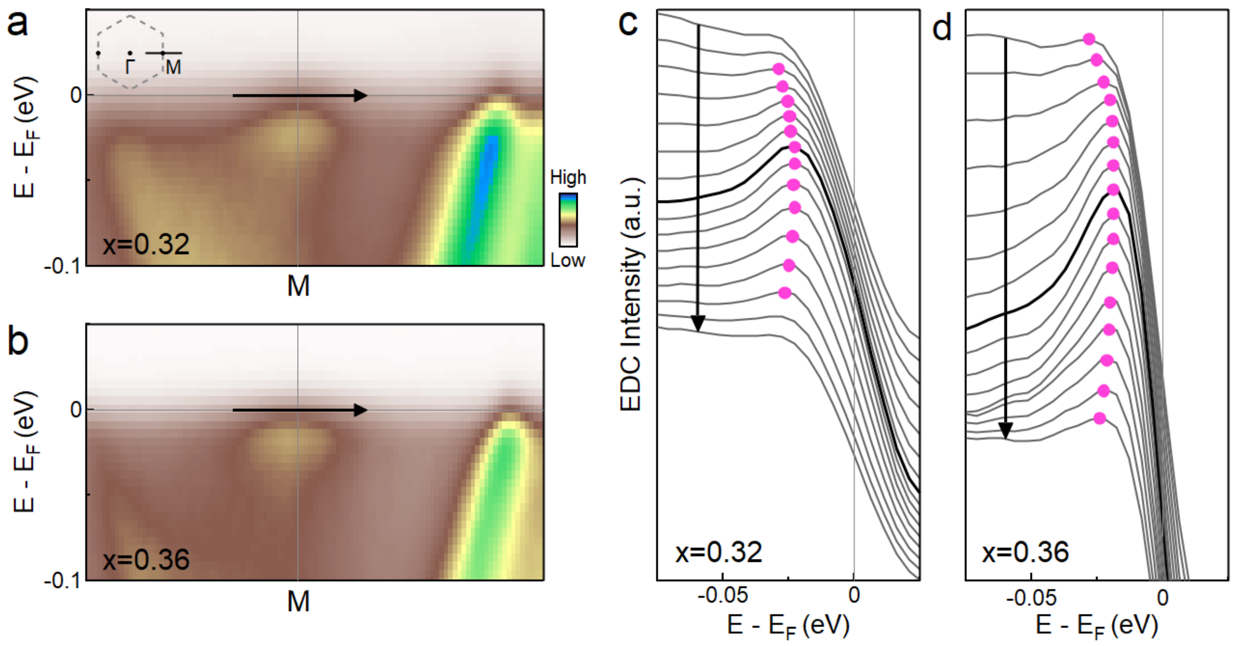

Fig. S 16: **Hole-like dispersion of the δ band along the Γ-M direction. a-b** Band structures of $Co_{0.32}TaS_2$ (**a**) and $Co_{0.36}TaS_2$ (**b**) along the Γ-M direction, as indicated by the black line in the inset of (**a**). **c-d** EDC stacks extracted along Γ-M, as marked by the arrows in (**a**) and (**b**), respectively. The peak positions are marked by dots.

[1] L. Xie, et al. Structure and Magnetism of Iron- and Chromium-Intercalated Niobium and Tantalum Disulfides. *Journal of the American Chemical Society*, **144**: 9525-9542, 2022.

[2] L. Xie, et al. Comparative Electronic Structures of the Chiral Helimagnets $Cr_{1/3}NbS_2$ and $Cr_{1/3}TaS_2$.. *Chemistry of Materials*, **35**: 7239-7251, 2023.

[3] A. Damascelli, et al. Angle-Resolved Photoemission Studies of the Cuprate Superconductors.. *Reviews of Modern Physics*, **75**: 473-541, 2003.
